# *In Silico* Modeling of Human α_2C_-Adrenoreceptor Interaction with Filamin-2

**DOI:** 10.1371/journal.pone.0103099

**Published:** 2014-08-11

**Authors:** Marcin Pawlowski, Saras Saraswathi, Hanaa K. B. Motawea, Maqsood A. Chotani, Andrzej Kloczkowski

**Affiliations:** 1 Battelle Center for Mathematical Medicine, The Research Institute at Nationwide Children's Hospital, Columbus, Ohio, United States of America; 2 Center for Cardiovascular and Pulmonary Research, The Research Institute at Nationwide Children's Hospital, Columbus, Ohio, United States of America; 3 Department of Pharmacology & Toxicology, Helwan University, Helwan, Egypt; 4 Department of Pediatrics, The Ohio State University, Columbus, Ohio, United States of America; Temple University School of Medicine, United States of America

## Abstract

Vascular smooth muscle α_2C_-adrenoceptors (α_2C_-ARs) mediate vasoconstriction of small blood vessels, especially arterioles. Studies of endogenous receptors in human arteriolar smooth muscle cells (referred to as microVSM) and transiently transfected receptors in heterologous HEK293 cells show that the α_2C_-ARs are perinuclear receptors that translocate to the cell surface under cellular stress and elicit a biological response. Recent studies in microVSM unraveled a crucial role of Rap1A-Rho-ROCK-F-actin pathways in receptor translocation, and identified protein-protein interaction of α_2C_-ARs with the actin binding protein filamin-2 as an essential step in the process. To better understand the molecular nature and specificity of this interaction, in this study, we constructed comparative models of human α_2C_-AR and human filamin-2 proteins. Finally, we performed *in silico* protein-protein docking to provide a structural platform for the investigation of human α_2C_-AR and filamin-2 interactions. We found that electrostatic interactions seem to play a key role in this complex formation which manifests in interactions between the C-terminal arginines of α_2C_-ARs (particularly R454 and R456) and negatively charged residues from filamin-2 region between residues 1979 and 2206. Phylogenetic and sequence analysis showed that these interactions have evolved in warm-blooded animals.

## Introduction

The α_2_-adrenoceptors (α_2_-ARs) are members of the G protein-coupled family of receptors (GPCRs), which is one of the largest families of proteins in the human genome [1], [2]. GPCRs are known to mediate important physiological functions and therefore, are targets for many current drugs; It has been estimated that 30% of major drugs target these receptors [3]. Three human α_2_-AR subtypes have been cloned and designated α2C10, α2C2 and α2C4 based on their human chromosomal localization, and subsequently renamed to α_2A_-ARs, α_2B_-ARs and α_2C_-ARs, respectively [4]. Phylogenetic classification of α_2_-ARs shows that they belong to the biogenic amine receptor cluster of the α-Group of Rhodopsin receptors [5].

Within the three α_2_-ARs subtypes, α_2C_-ARs have unique regulation, cellular localization and trafficking profile in the human and murine microvasculature. The α_2C_-ARs modulate blood flow and are preferentially expressed in the smooth muscle cells of the microcirculation, particularly arterioles [6]. The α_2C_-ARs mediate vasoconstriction upon stimulation by the endogenous agonist norepinephrine (noradrenaline) and therefore, have a unique and crucial role in physiology and pathophysiology involving the peripheral circulation [7], [8]. It is therefore important to understand mechanisms of receptor expression and trafficking for a clear understanding of α_2C_-AR biology. The α2C-ARs are intracellular receptors that are known to translocate to the cell surface under stress conditions such as cold temperature. They play a vital role in skin thermoregulation [8], [9]. In heterologous HEK 293 cells, α2C-ARs are present in the transGolgi at physiological 37°C temperature. Moderate cooling to 28°C leads to cell surface translocation of functional α2C-ARs [10]. The mechanism of cooling-triggered translocation involves release of mitochondrial reactive oxygen species, activation of RhoA-ROCK signaling, and receptor cell surface translocation [11], [12]. Recent studies have identified a temperature-independent (i.e. physiological 37°C coupled), and cyclic AMP (cAMP)-dependent mechanism of receptor expression and translocation coupled to the cAMP receptor Epac and Rap1A-Rho-ROCK signaling pathway [13]. Cyclic AMP leads to increased transcription of α_2C_-ARs through JNK-c-jun nuclear signaling and increased cell surface translocation of mature receptors through RhoA-ROCK signaling and F-actin coupled pathway [13], [14]. Therefore, divergent signaling pathways, including cooling-triggered or cAMP-triggered converge on a common pathway, are necessary for receptor translocation to the cell surface.

More recent studies have identified protein-protein interaction between the α_2C_-AR carboxyl terminus and the actin-binding protein filamin-2 in mediating cell surface translocation of intracellular receptors [15]. In this study we performed computational modeling of α_2C_-AR to filamin-2 binding in order to better understand protein-protein specificity of this interaction. Our studies show that this approach complements and supports the experimental approaches utilized in previous studies [15].

## Materials and Methods

### Sequence analyses

#### α2C-adrenoceptors (ADRA2C)

Searches of human α_2C_-adrenoceptor homologs were carried out using a locally installed version of PSI-BLAST algorithm [16] against the non-redundant (nr) version of the NCBI sequence database (as of June, 2014). The gapped blast algorithm (blastpgp) with the expectation value (E-value) threshold for the retrieval of related sequences set to 0.001. Three iterations of PSI-BLAST were run, and all sequences from hits with an expectation value better than 0.001 were retrieved.

#### Sequence clustering

α_2C_-adrenoceptors belong to a large family of G protein-coupled receptors [1], [2]. Hence, a homology search, yielded 72,730 proteins. To facilitate further analysis, we applied sequence clustering using CLANS [17], to group these sequences into families. CLANS (Cluster ANalysis of Sequences), is a Java program that applies a version of the Fruchterman-Reingold graph layout algorithm for visualizing protein families based on pairwise similarity. This algorithm helps to represent the force between any two nodes, where each node represents a pair of proteins. In order to draw graphs in an aesthetically pleasing way, the algorithm has to minimize the energy of the system by moving the nodes and changing the forces between them. CLANS uses the P-values of high-scoring segment pairs (HSPs) obtained from an N×N BLAST search, to compute attractive and repulsive forces between each sequence pair in a user-defined dataset. Two or three dimensional representation is achieved by randomly seeding sequences in space. The sequences are then moved within this environment according to the force vectors resulting from all pairwise interactions and the process is repeated to achieve convergence.

#### Multiple Sequence alignment

All sequences classified as members of the α_2_-adrenoreceptor superfamily were aligned using MUSCLE [18]. Incomplete sequences were discarded (if the deletion spanned >30% of the alignment). BioEdit program [19] was used to manually optimize the alignments to preserve the continuity of secondary structure elements, including transmembrane helices.

#### Phylogenetic analyses

The phylogenetic tree of the α_2_-adrenoceptor superfamily was inferred for all members of this family. Based on the multiple sequence alignment provided by MUSCLE, MEGA 5 [20] was used to construct a minimum evolution phylogenetic tree, with pairwise gaps deletion and JTT matrices [21]. The stability of individual nodes was calculated using the bootstrap test (with 100 replicates) and additionally confirmed by the interior branch test (IBT).

### Protein structure prediction

Human α_2C_-adrenoceptor and has been experimentally shown to interact with the filamin-2 (FLN2) region [15]. In the absence of experimentally determined structure that shows this interaction, we constructed comparative models of human FLN2 protein (gi number: 8885790) and human ADRA2C protein (GI number: 3914602). In the following sections, structure prediction of these two proteins are discussed in detail.

#### Modeling of filamin-2 (FLN2) region

According to the HHpred [22] program, three protein domains were found within the FLN2 fragment that had been shown to be responsible for ADRA2C binding [15]. Based on the predicted domain boundaries we redefined the filamin-2 region that binds ADRA2C, to 202 amino acid residues that are located between residue 1982 and 2183. This region was investigated by using the state-of-the art structure prediction servers, that include GeneSilico metaserver [23], Zhang-Server [24], Robetta [25], HHpred [22], and Multicom [26] server. The initial models provided by these servers were submitted to the QA-RecombineIt server [27], which operates in two stages. In the first stage, the server predicts both global and local accuracy of models. In the second stage, the server runs an algorithm that performs a ‘recombination’ of the best ranked parts of the input models into new hybrid structures that are likely to be better than the input models themselves. By using recombination of the initial models, the QA-RecombineIt generated 100 additional consensus models. From these models, the final model was selected by using Model Quality Assessment Programs, such as MetaMQAP [28], ProQ2 [29], GOAP [30], DFIRE [31] and MQAPmulti (M Pawlowski, unpubl.).

#### Modeling of α2C-adrenoceptor

To model the structure of human α_2C_-adrenoceptor, its sequence was submitted to GeneSilico metaserver [23], Zhang-Server [24], Robetta [25], HHpred [22], and Multicom [26] server. Noteworthy, in contrast to FLN2 protein, the α_2C_-adrenoceptor is a transmembrane protein. Hence, in addition to these aforementioned protein structure prediction servers, we used servers optimized to predict 3D structure of transmembrane proteins. Among these servers were: GPCRM [32], GPCR-ITASSER [33] and GPCR-SSFE [34]. These servers created 145 initial models in total, which were used as input for the QA-RecombineIt server [27]. By using recombination of the initial models, the QA-RecombineIt generated 100 additional consensus models. From these models, the final model was selected by using MQAPmulti (M Pawlowski, unpubl.). and ProQM [35] programs. Notably, ProQM is the only one MQAP that has been created to predict the correctness of transmembrane proteins.

### Docking between ADRA2C and FLN2 region between amino acid residues 1982 and 2183

Docking models of the ADRA2C and FLN2 complex were generated with the HADDOCK server [36], [37]. Docking by HADDOCK is driven by predictions of likely residues involved in protein-protein interface (ambiguous interaction restraints (AIRs)). Such residues may be active (interacting residue) or passive (solvent-accessible neighbor of interacting residue). AIRs for both ADRA2C and FLN2 (residues 1982 to 2183) were predicted by using CPORT [38]. Then, two hundred complexes were generated by the HADDOCK program and clustered. Selection of the best complex was based on cluster size, HADDOCK score and electrostatic energy. Among the ten best clusters, we selected a cluster that was the second most populated cluster, but was characterized by highest HADDOCK score and lowest electrostatic energy. PISA [39] was used to analyze the protein-protein docking and binding interfaces. The illustrations and visualizations of the final model were produced in PyMOL (version 1.4.1) [40].

## Results

### Sequence database searches and retrieval of members of the α_2_-adrenoceptor family

To identify a complete set of α_2C_-adrenoceptor sequences, including sequences of α_2A_-, α_2B_-, and α_2C_-adrenoceptors, we used full-length sequences of representatives of these three types of α_2_-adrenoceptors (GI numbers: 194353970, 33598960 and 3914602) from *H.sapiens* to carry out homology search. We removed identical proteins retrieved in different searches and finally, we obtained 72,730 proteins homologous to the α_2_-adrenoceptors. Notable, all members of α_2_-adrenoceptors were find during the first iteration of PSI-BLAST.

### Extracting α_2_-adrenoceptors from G protein-coupled receptors

**Figure 1:**
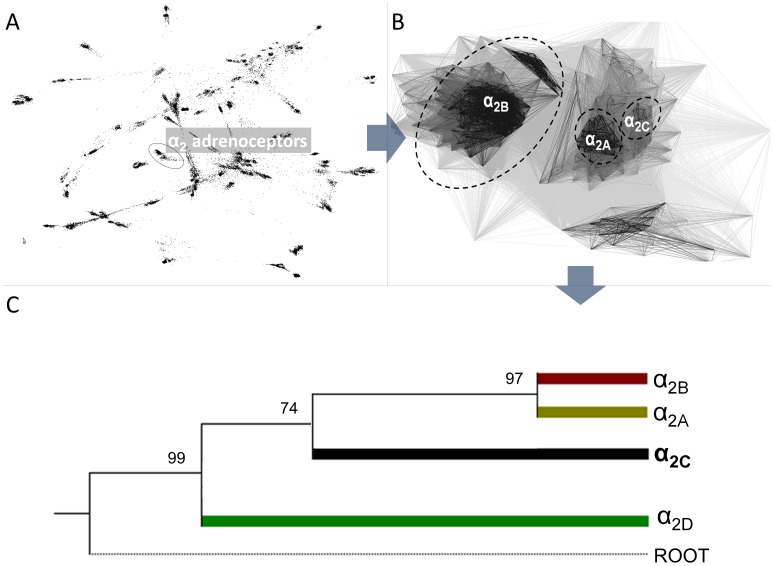
Initial clustering of GPCR proteins and phylogenetic tree of all α_2_-adrenoceptors. Panel A presents two-dimensional projection of the CLANS clustering results obtained for the GPCR proteins, a clan corresponding to α_2_-adrenoceptors is indicated by an ellipse. Panel B presents two-dimensional projection of the CLANS clustering of α_2_-adrenoceptors. Panel C presents the postulated phylogenetic tree of the α_2_-adrenoceptor family. Only the major branches corresponding to subfamilies are shown. Values at the nodes indicate the statistical support for the particular branches, according to the bootstrap test. The human rhodopsin sequence was used for rooting the tree.

Since the performed homology searches had provided not only the α_2_-adrenoceptors, but also other G protein-coupled receptors (GPCRs), we performed the clustering of these all proteins to identify clusters that contain α_2_-adrenoceptors only. We clustered the G protein-coupled receptors based on the pair-wise BLAST similarity scores by using the CLANS program [17]. We tried different P-value thresholds and found that the value of 10^−11^ produced best resolved sequence “clans” corresponding to different highly homologous subtypes of α_2_-adrenoceptors, including α_2A_-, α_2B_-, and α_2C_-adrenergic (with strong connections within each clan and preferred connections between a few, but not all clans) (Figure 1, panel A). Figure 1, panel B focuses only on α_2_-adrenoceptor family. Even though, fraction of α_2_-adrenoceptor proteins were clustered clearly as one of the α_2_-adrenoceptor subfamilies, the classification of some α_2_-adrenoceptors was still unsolved.

### Multiple sequence alignment and phylogenetic analysis of α2-adrenoceptors

Based on the results of preliminary clustering, we extracted only members of α_2_-adrenoceptor family. Then we calculated family-specific multiple sequence alignments using MUSCLE [18] and adjusted them manually (as described in Methods) to remove truncated sequences and redundant, nearly identical versions of the same protein, and to improve the placement of insertions and deletions. The refined multiple sequence alignment was used to infer the phylogenetic tree of the α_2_-adrenoceptor family by using Minimum Evolution (ME) analysis carried out with MEGA 5 [20]. To calculate the stability of individual nodes, the bootstrap test and the interior branch test were applied. Noteworthy, for this phylogenetic tree, for all branches with bootstrap support >50%, the ITB support was equal or higher 50.

The Minimum Evolution tree (Figure 1, panel C) provides significant support for main branches, allowing us to resolve the deep branching pattern. This compressed tree indicates the division of α_2_-adrenoceptors family into 4 subfamilies. This approach clearly showed that the α_2C_-ARs are relatively distinct and form a separate branch, while the α_2A_-ARs and α_2B_-ARs are the most closely related to one another. These mutual orientations of the α_2A_-AR, α_2B_-ARs and α_2C_-ARs are in agreement with the previously published phylogenetic analysis of these subtypes [41]. The tree also supports the findings of Ruuskanen et al that there is also another subtype of α_2_-adrenoceptors, named α_2D_-adrenoceptors, and originally identified in Zebrafish by comparison of ligand binding characteristics of α_2_-adrenoceptors, but not by phylogenetic analysis [42]. The α_2D_-adrenoceptors form the separate branch showing that this family is relatively distinct from other subfamilies. Noteworthy, since the rodent orthologue of the human α_2A_ is occasionally misleadingly called α_2D_, we want to emphasize that in our work we follow the naming proposed by Ruuskanen et al.

**Figure 2:**
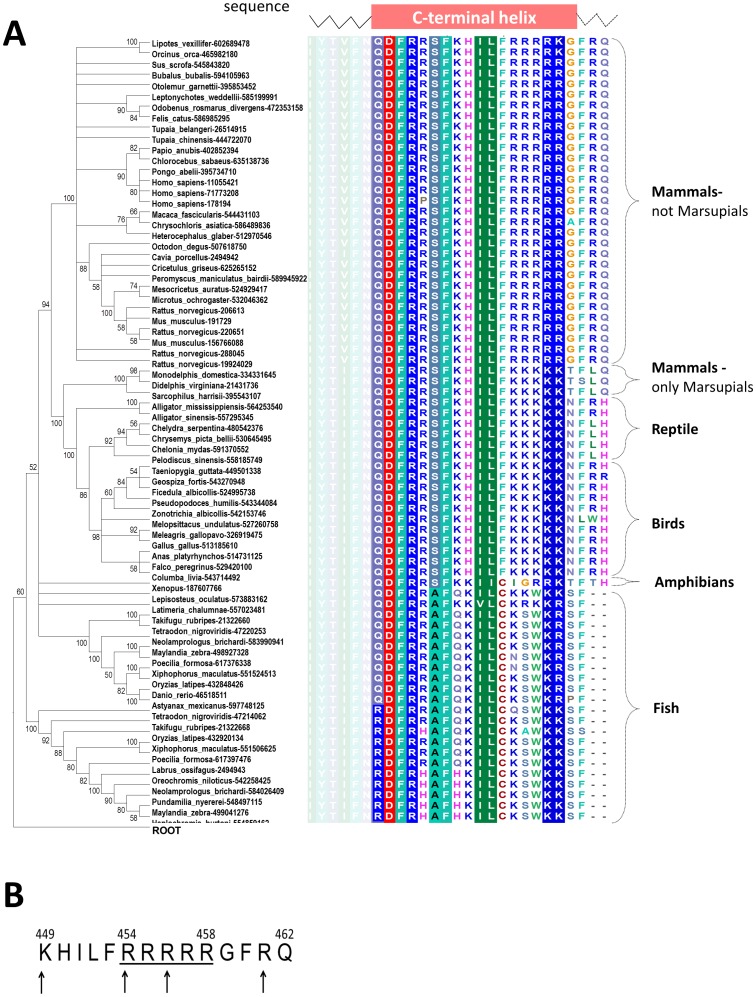
The Minimum evolution tree and multiple sequence alignment of C-terminal tail of the α_2C_-adrenoceptor family. Panel A - proteins are indicated by the species name and the NCBI GI number. Values at the nodes indicate the statistical support for the particular branches, according to the bootstrap test. For each protein also its C-terminal sequence is presented. Sequences were aligned by MUSCLE program. Amino acids are colored according to the chemical properties of their side-chains (negatively charged: red, positively charged: blue, polar: magenta, hydrophobic: green. Only the alignment that corresponds to the C-terminal helix and flanking residues is shown. The helix was predicted by GeneSilico metaserver. Panel B - the last 14 amino acids of a2C-AR C-terminus highlighting the arginine-rich stretch (underlined). This region is conserved in mammals and in human arteriole-derived vascular smooth muscle cells (microVSM) interacts with the actin-binding protein filamin-2, shown in experimental studies to be necessary for receptor translocation to the cell surface. The numbers denote amino acids in the full-length a2C-AR polypeptide. The arrows point to amino acid residues identified by *in-silico* modeling to be involved in interaction with filamin-2.

Detailed analysis of the evolution of the C-terminus of α_2C_-adrenoceptors is presented in Figure 2, panel A. The α_2C_-adrenoceptors appears in nearly all sequenced Vertebrata. The α_2C_-adrenoceptors are divided into 5 subfamilies containing members found in: 1) Mammals, excluding Marsupials, 2) only Marsupials, 3) Birds, 4) Amphibians, 5) Reptile, and 6) Fish. Interestingly, the C-terminus of α_2C_-adrenoceptors is either Arginine- or Lysine-rich only in Mammals and Birds. This finding may be connected with the fact that these warm-blooded animals need systems to control temperature of the most peripheral parts of their bodies. We postulate that the α_2C_-adrenoceptor may be involved in the process, in which the receptor's highly positively charged C-terminal helix may be responsible for the receptor translocation.

To investigate this hypothesis, we decided to build a computational model of the complex of α_2C_-adrenoceptor and filamin-2, which is presented in the following paragraphs.

### Computational simulation of α_2C_-AR-filamin-2 binding domains

We performed computational modeling predictions of full-length α_2C_-AR and filamin-2 (amino acids 1979–2206) structure to better understand the specificity of α_2C_-AR-filamin-2 protein-protein interactions. In the absence of a crystal structure for α_2C_-AR and filamin-2 region, we utilized amino acid homology searches, domain predictions, followed by protein-protein docking, to identify the residues that play a key role in α_2C_-AR-filamin-2 recognition and binding as described below.

#### Modeling of filamin-2

**Figure 3:**
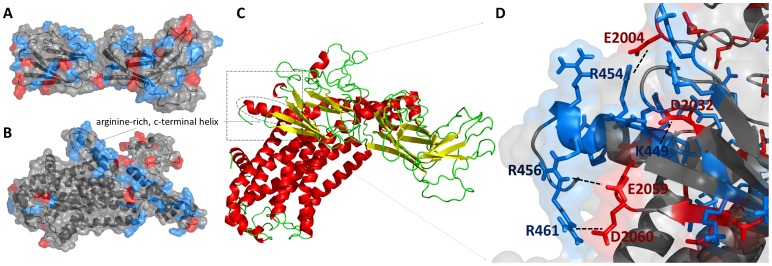
Predicted models of filamin-2 (FLN2) and α_2C_-adrenoceptor (ADRA2C) proteins, and their complex. Panels A and B present cartoon diagram of FLN2 (region between residues 1982 and 2183) and ADRA2C protein models. Positively and negatively charged regions are indicated by blue and red colors, respectively. Panel C presents whole protein-protein complex predicted by HADDOCK program. Panel D shows the interaction between receptor's C-terminal helix and the filamin-2 region that is responsible for binding the receptor.

In the absence of experimentally determined structure for functionally characterized human filamin-2, we constructed a comparative model of a human filamin-2 region (amino acids 1979–2206) found to bind α_2C_-adrenoceptor. First, to perform initial sequence analysis the sequence of FLN2 (amino acids 1979–2206) was submitted to GeneSilico metaserver [23]. This analysis revealed that this region is composed of three domains (roughly residues 1982–2100, 2101–2178 and 2179–2183). Both the N-terminal and C-terminal domains of FLN2 were found to exhibit significant similarity to Filamin/ABP280 repeat family, whose members have been found to interact with such proteins like: β-Integrin, Rho, Rho-associated kinase (ROCK), and many others [43]. In contrast to the N-terminal and C-terminal domains of FLN2, the domain in the middle (2101–2178 residues) exhibited no evident similarity to any known protein family. Nearly all individual fold-recognition methods (e.g., HHSEARCH: score 100, FFAS score -50.1, COMPASS score: 2.72e-59, PHYRE score: 1e-19) reported the structure of the protein with PDB code 2j3s [crystal structure of filamin A Ig domains 19–21] as the potentially best template to model FLN2 region 1982–2183 (i.e. its closest homologs among proteins of known structure); the sequences of 2j3s and the target proteins share 54% sequence identity. In the next step, the sequence of FLN2 (amino acids 1979–2206) was submitted also to Zhang-Server [24], Robetta Server [25], HHpred [22], and Multicom [26] server; these servers have been shown to be the best automatic methods for proteins structure modeling [44]. In total we collected 145 initial models, which were submitted to the QA-RecombineIT [27] server that operates through following two stages. In the first stage (QA-mode), the server predicts both the global quality of input models and provides estimates of local quality as the deviation between C-α atoms in the models and corresponding atoms in the unknown native structure. The input models and the predictions of the models' correctness become the input for the second stage (RecombineIt-mode), in which fragments predicted to be better than others are judiciously combined to generate hybrid (consensus) models. Finally, hybrid models are scored by the MQAPs implemented in the QA-mode and then presented to the user. By using recombination of the initial models, the QA-RecombineIt generated 100 additional consensus models for the filamin-2 region between residue 1982 and 2183. From these models, the final model was selected by using Model Quality Assessment Programs, such as MetaMQAP [28], ProQ2 [29], and MQAPmulti (M Pawlowski, unpubl.). These methods predict GDT_TS score of a protein model without the knowledge about the true native structure of the protein. Global Distance Test (GDT_TS) corresponds to the average value of fractions of C-α atoms in the model that are placed within the distances of 1, 2, 4 or 8 Å from corresponding C-α atoms in the experimentally determined structure. This metric has values in the [0,1] range, where 1 corresponds to the highest quality model. In contrast to RMSD (root-mean-square-deviation) score, GDT_TS-score is insensitive to local structure variation. In general, two structures with GDT_TS-score lower than 0.3 correspond to random similarity and those with GDT_TS-score at least 0.5 indicate high similarity between the predicted model and native structure. Model Quality Assessment Programs, may be divided into two main classes: 1) single-model MQAPs - methods capable of assessing quality for single models, without using any alternative models (decoys) generated for the same protein; 2) clustering MQAPs – methods that perform all against- all structural comparisons to obtain mean similarity scores for ranking models. Moreover, it was shown that a linear combination of scores provided by clustering and single model MQAP perform well for selection of the most accurate model from a set of alternative models for the target protein [29]. Thus, to select the final model of filamin-2 region (amino acids 1979–2206) we applied a linear combination of MQAPmulti (a clustering MQAP, weight: 0.8) and two single model MQAPs MetaMQAP, and ProQ2 (weight: 0.1 each), then the model with the highest score was selected as the final model. The selection procedure was inspired by the findings that a linear combination of scores provided by clustering and single model MQAPs perform well for selection of the most accurate model from a set of alternative models for the target protein [29]. For the best-scoring structure the MetaMQAP, ProQ2 and MQAPmulti GDT_TS scores were as follows, 0.51, 0.43 and 0.78. This model is presented in Fig. 3 panel A.

#### Modeling of α2C-adrenoceptor

Similar to the modeling approach used for filamin-2 we performed initial sequence analysis of α_2C_-adrenoceptor by using GeneSilico metaserver. As expected, α_2C_-adrenoceptor, a G protein coupled receptor, has been predicted to have 7 transmembrane helices. FR algorithms suggested that the potentially best templates for modeling of α_2C_-adrenoceptor are either human M2 muscarinic acetylcholine receptor (pdbcode: 3uon) or human A2A adenosine receptor (pdbcode: 3eml), proteins sharing 22% and 20% sequence similarity to the target, respectively. Since α_2C_-adrenoceptor is a transmembrane protein, we submitted the α_2C_-adrenoceptor sequence to Zhang-Server, Robetta Server, HHpred, and Multicome servers (methods devoted to predict structure of globular proteins) in the second step. We also used other servers that were created and optimized to predict 3D structure of transmembrane proteins such as GPCRM [32], GPCR-ITASSER [33], and SSFE [34]. Including models provided by the GeneSilico server, we collected 167 models for the α_2C_-adrenoceptor proteins. Next, these models were submitted to QA-RecombineIt method for model quality assessment and models recombination. Once the QA-RecombineIt generated additional 100 hybrid models, the models were combined with the 167 input models, and the best-scoring model was selected. As a criterion the sum of MQAPmulti (M Pawlowski, unpubl.) and ProQM [35] scores were used. Noteworthy, in contrast to Filamin2, α_2C_-adrenoceptor is a transmembrane protein, thus we had to use different combinations of MQAPs to select the best models. Here, we used MQAPmulti, a method that belongs to clustering MQAPs, which was shown to perform well for transmembrane proteins [45], and ProQM [35], which is a variant of the ProQ2 method [29], but was devoted to predict the accuracy of models built for transmembrane-proteins. Then, in analogy to what we did during the modeling of filamin-2, we applied a linear combination of MQAPmulti (a clustering MQAP, weight: 0.8) and ProQM (weight: 0.2) to select the final model. For the best-scoring model the ProQM and MQAPmulti GDT_TS scores were as follows, 0.609, 0.76, which showed an improvement compared to the best model from the 167 initial models having ProQM and MQAPmulti scores of 0.580, and 0.87. This model is presented in Fig. 3 panel B.

#### Docking between ADRA2C and FLN2 region between amino acid residues 1982 and 2183

Docking models of the ADRA2C and FLN2 complexes were generated with HADDOCK webserver, using the 3D structures previously created for human α_2C_-adrenoceptor and Filamin-2 (residues 1982–2183). Due to the lack of experimental data about possible structure of the complex, the AIRs for both ADRA2C and FLN2 region between residues 1982 and 2183 were predicted by using the CPORT algorithm. Such a combination of CPORT and HADDOCK has performed well for cases where no experimental data were available [38]. As the procedure was described by the HADDOC authors, the first docking step consisted in a rigid body energy minimization. After this step, 500 best solutions were selected for 3 rounds of simulated annealing refinements including: 1) rigid bodies optimization of mutual orientation of the two proteins, 2) side chains refinement at the interface, and 3) side-chain and backbone optimization at the interface between these two proteins. Finally, 200 complexes with the highest scores were clustered. The resulting clusters were analyzed and ranked according to the HADDOCK score which consists of a weighted sum of energies that include intermolecular electrostatic, van de Waals, desolvation and AIR (ambiguous interaction restraints) and a buried surface area term. HADDOCK clustered 146 structures in 10 cluster(s), which represents 73.0% of the water-refined models HADDOCK generated. The largest cluster had 41 structures, the 5^th^ best HADDOCK score (−91.2) and the 6^th^ best (lowest) electrostatic energy (−508.8) among all 10 clusters. However, the protein-protein interfaces among those structures did not involve any interactions between the C-terminal helix of *ADRA2C* and FLN2 between residues 1982 and 2183, as previously proven to occur by Motawea and coworkers [15]. Thus, among the ten most populated clusters, we searched for clusters that had receptor-filamin complexes having C-terminal helix of the receptor molecule involved in interactions with the filamin molecule. Cluster number 7 was the only one that satisfied this criterion. This cluster was the 7^th^ most populated one (8 members), had the 4^th^ highest HADDOCK score (−126.8), but was characterized by the best electrostatic energy among all clusters (−938.1). The medoid of this cluster was selected as the final solution of protein-protein docking.

#### Protein-protein interface between ADRA2C and FLN2 region between amino acid residues 1982 and 2183

Figure 3 panel D presents the protein-protein interface most likely to be involved in the recognition and binding of α_2C_-adrenoceptor by human filamin-2. The interface area, measured by PISA server [39], occupied 1277.6 Å^2^. Three arginines (R454, R456 and R461) are stabilized by negatively charged residues in the filamin-2 structure: E2004, E2059 and D2060, respectively. Another interaction involved in the complex stabilization is lysine K449 that is stabilized by aspartic acid at position 2032 (D2032) in the filamin-2 sequence.

Multiple sequence alignments of ADRA2C (presented in Figure 2, panel A) and FLN2 (region between amino acid residues 1982 and 2183, no data shown) reveals that the residues found to be involved in the complex stabilization are conserved between the homologs of human ADRA2C and FLN2, which is typical for protein–protein interaction sites [46]. Noteworthy, in the case of ADRA2C the conservation is observed only for Mammals and Birds, that is in contrast to that observed for FLN2, where the conservation is observed for all members of this family. Taken together, these findings suggest that these two genes have not coevolved, but the genes of ADRA2C animals have evolved in order to interact with the filamin-2 in warm-blooded animals.

## Discussion

Recently it was shown that the C-terminal helix of human α_2C_-adrenoceptor binds to filamin-2 region between residues 1979 and 2206 [15]. To study this interaction in the context of evolution, we have carried out extensive bioinformatics analyses and proposed a structural model of this complex. The approach used in the present study complements and supports the experimental approach described in the previous study [15].The results of multiple sequence alignment of α_2C_-adrenoceptor family combined with the phylogenetic analysis showed that among all animals studied here, only the warm-blooded ones have α_2C_-adrenoceptor C-termini that are either Arginine- or Lysine-rich. We postulate that this highly positively charged helix is involved in the binding of the α_2C_-adrenoceptor to the filamin-2, in which satisfying the electrostatic energy is the driving force. The last 14 amino acids, unique to the α_2C_-AR subtype (Figure 2, panel B), play a critical role in protein-protein interaction with filamin-2. Such binding, as shown by Motawea et al., 2013 [15] is responsible for translocation of functional receptors to the cell surface. Motawea et al 2013 also performed site-directed mutagenesis of the arginine-rich region (R454 to R458) and replaced all five arginines with non-polar alanines (A454 to A458). The receptor with these alanines was not able to interact with filamin-2. This finding, together with the fact that the α_2C_-adrenoceptors with a non-positively charged C-terminal helices occur only in cold-blooded animals, supports our postulate that the positive charge is critical for the binding and translocation. The molecular docking reveals the interactions involved in the creation of the protein-protein interface between these two proteins, particularly R454 and R456 in stabilizing this interaction. We found that there are four interactions that stabilize the positive charge of the C-terminal helix, including three arginines (R454, R456 and R461) that are stabilized by negatively charged residues in the filamin-2 structure: E2004, E2059 and D2060, respectively. Another interaction involves lysine 449 (K449) that is stabilized by aspartic acid at position 2032 (D2032) in filamin-2. We postulate also, that the arginines numbered as R455, R457 and R458 are also important for the creation of the protein-protein interface, although they were not shown by the protein-protein docking study (see Fig. 3) to create any important interactions within the protein-protein interface. However, they can act as O-ring residues [47] whose role is to occlude bulk water molecules from the hot spots. Exclusion of water from the binding interface is thought to be entropically favorable. In addition, removing of solvent dipoles lowers the local dielectric constant for the hotspot, increasing the energetic contribution of electrostatic interactions [47].

Indeed, experimental studies performed by Motawea et al show that the receptor having arginines (R454–R458) replaced with alanines (A454–A458) does not associate with filamin-2 [15]. Experimental studies also suggest the role of the arginine-rich region (R454–R458) in retaining mature receptors in the Golgi compartment. In transiently transfected HEK293 cells the mature glycosylated receptor (the ∼70 kDa form that has passed through the ER, cis/medial Golgi and is endoglycosidase H resistant) is retained in the *trans*Golgi, and translocates to the cell surface in response to stimulus including cold temperature [10]. The receptor having arginines replaced with alanines however, is no longer retained and is localized on the cell surface [15]. The studies therefore suggest that α_2C_-AR interaction with filamin-2 enables stimulus-dependent regulated cell surface delivery and function compared with constitutive presence on the cell surface.

It remains to be determined why the C-terminal helix is arginine-rich in Mammals (not including Marsupials) and lysine-rich in the rest of warm-blooded animals. As shown in figure 2, panel A, the C-terminal helices of the α_2C_-ARs *in* Fish are both lysine- and arginine-rich. It may suggest that in the common ancestor of all warm-blooded animals the α_2C_-AR could have had both arginine and lysine rich C-terminal helix, and during the species speciation the lysine-rich variant has been kept among Birds and Marsupials, in contrast to the arginine-rich variant that has been kept among the rest of Mammals. Taking this hypothesis into account, it would be interesting to see what will happen if the human α_2C_-AR has its C-terminal helix replaced by the Birds/Marsupials lysine-rich variant. Could it function the same way as the wild-type variant of the receptor in skin thermoregulation in humans? Future experimental studies will allow examination of this hypothesis.

It has been shown that α_2C_-ARs are intracellular receptors that are translocated to the cell surface in response to cellular stress including cold temperatures and play a vital role in skin thermoregulation [8], [9]. However, it is estimated that in 5–10% of the general US population, this system is overregulated and leads to Raynaud's phenomenon, an exaggerated vasospastic response to cold or to emotional stress [48]–[50]. One of the possible attempts to find therapeutics for Raynaud's would be to develop small molecules that are antagonists of human α_2C_-ARs [51]. However, since it has been shown that α_2C_-ARs are responsible for, among others, sympathetic neurotransmission - [52], the usage of such an antagonist would be likely to be associated with many serious side-effects in humans. Notable, in the present study we showed the possible interface between the α_2C_-AR and filamin-2. We believe that this finding may contribute to the development of new therapeutics for Raynaud's phenomenon that target the protein-protein interface between those two proteins, selectively inhibiting cell surface translocation of intracellular receptors. Our optimism is based on the fact that targeting protein-protein interface has been very successful in drug design, for example in identifying inhibitors of the Bcl-2 protein [53] or inhibitors of the binding of S100B, a calcium binding protein, and p53 [54]. We believe that in the case of Raynaud's syndrome such therapeutics can target the protein-protein interface between the filamin-2 and intracellular α_2C_-AR, but spare surface receptors expressed in other tissues, reducing side-effects. It is also interesting if targeting the drug design towards the protein-protein interface instead of α_2C_-ARs itself would help to avoid some issues associated with recent GPCR drug discovery. One of these issues arises from the observation that many of possible small molecules that target GPCRs, but not the protein-protein interface in which GPCRs are involved, are generally at the upper limits of Lipinski's rules in terms of molecular weight and/or lipophilicity [55], [56]. This suggests that they would have been “high risk” in terms of both toxicity and cross-reactivity giving a low success rate in the clinic [57], [58]. Thus, we hope that drugs interfering with the protein-protein interface of α_2C_-ARs and filamin-2 will be less prone to these negative side effects.

## Conclusions

Here, we showed extensive bioinformatics analyses aimed to study the binding of α_2C_-adrenoceptor to filamin-2 region between residues 1979 and 2206, which has lead us to the following findings and conclusions. First, by protein-protein docking, we characterized the protein-protein interface, in which the C-terminal helix of α_2C_-adrenoceptor is involved in the complex creation. Second, the electrostatic interactions seem to play a key role in this complex formation which manifests in interactions between the C-terminal arginines of α_2C_-ARs (particularly R454 and R456) and negatively charged residues from filamin-2 region between residues 1979 and 2206. Finally, multiple sequence alignments and phylogenetic analysis showed that these interactions are conserved in warm-blooded animals.

According to the 3did database [59], a catalog of domain-based interactions of known three-dimensional structure, there is no crystal structure where the C-terminal helix of a GPCR protein was involved in protein-protein interface. Thus, we believe that this model of the α_2C_-adrenoceptor-filamin-2 complex will help in the further investigation of the mechanism of the GPCR protein translocation to any cell compartment, including the α_2C_-adrenoceptor translocation to the cell surface in the context of cellular physiology and pathophysiology.
